# Dyslipidemia among Patients with Ischemic Stroke Admitted to the Department of Medicine of a Tertiary Care Centre

**DOI:** 10.31729/jnma.8261

**Published:** 2023-09-30

**Authors:** Tunam Khadka, Ganesh Kumar Giri, Pasang Sherpa, Urbi Ghimire, Shamin Parajuli, Ashok Pudasaini, Sajan Shrestha

**Affiliations:** 1 Kathmandu Medical College and Teaching Hospital, Sinamangal, Kathmandu, Nepal; 2 Department of Medicine, Nepal Medical College and Teaching Hospital, Jorpati, Kathmandu, Nepal; 3 Department of Medicine, Upendra Devkota Memorial National Institute of Neurological and Allied Sciences, Bansbari, Kathmandu, Nepal

**Keywords:** *cross-sectional studies*, *dyslipidemia*, *hyperlipidemias*, *ischemic stroke*

## Abstract

**Introduction:**

Sudden neurological deficits due to an acute focal vascular injury of the centra! nervous system is stroke. It is a major cause of morbidity and mortality. Dyslipidemia is any abnormality in the parameter of the lipid profile which has been associated with stroke. The aim of this study was to find out the prevalence of dyslipidemia among patients with ischemic stroke in the Department of Medicine of a tertiary care centre.

**Methods:**

A descriptive cross-sectional study was conducted in a tertiary care centre among admitted ischemic stroke patients from 5 August 2022 to 14 March 2023 after obtaining ethical approval from the Institutional Review Committee. Overnight fasting blood samples were collected for lipid profile. Convenience sampling method was used. The point estimate was calculated at a 95% Confidence Interval.

**Results:**

Among 79 ischemic stroke patients, 42 (53.16%) (42.09-64.23, at 95% Confidence Interval) had dyslipidemia where high cholesterol was seen in 21 (50%), high triglyceride in 22 (52.38%), high low-density lipoprotein 14 (33.33%) and low high-density lipoprotein in 20 (47.61%).

**Conclusions:**

The prevalence of dyslipidemia among ischemic stroke patients was lower than the similar studies done in similar settings.

## INTRODUCTION

Stroke is a neurological deficit due to an acute focal vascular injury of the central nervous system including cerebral infarction, intracerebral haemorrhage, and subarachnoid haemorrhage, and is a major cause of morbidity and mortality worldwide.^1^ The majority of patient suffers from ischemic stroke rather than hemorrhagic.^2^ Based on disability-adjusted life years, stroke is one of the major causes of death and is among the top five diseases in Nepal.^3^ The mean age of stroke patients in Nepal is between 59 and 62 years, with males affected more frequently.^4^

Dyslipidemia seems to be a major abnormal finding in stroke patients.^2^ Dyslipidemia is a group of metabolic derangements characterised by any or combination of elevated low-density lipoprotein cholesterol, total cholesterol, triglycerides, and low high-density lipoprotein.^5^ There was a three-fold increase in the incidence of stroke in patients with dyslipidemia.^6^

The aim of this study was to find out the prevalence of dyslipidemia among patients with ischemic stroke in the Department of Medicine of a tertiary care centre.

## METHODS

This was a descriptive cross-sectional study conducted in the Department of Medicine of Kathmandu Medical College and Teaching Hospital, Sinamangal, Kathmandu, Nepal among the cases of ischemic stroke that had been diagnosed in the hospital between from 5 August 2022 to 14 March 2023. Ethical approval was obtained from the Institutional Review Committee of the same institute (Reference number: 4082022/02). The study included patients who had developed a sudden neurological deficit affecting a vascular territory, with sustained symptoms lasting more than 24 hours and evidence of a stroke on magnetic resonance imaging or non-contrast computed tomography scan, as confirmed by a neurologist. Patients who were discharged or referred to another hospital, and patients who did not undergo neuroimaging were excluded from this study. Convenience sampling method was used. The sample size was calculated using the following formula:


$$\begin{array}{ll} \text{n} & ={\text{Z}}^{2}\times \frac{\text{p}\times \text{q}}{{\text{e}}^{\text{2}}}\\ & =1.96^{2}\times \frac{0.80\times 0.20}{0.09^{2}}\\ & =76 \end{array}$$

Where,

n = minimum required sample size
Z = 1.96 at 95% Confidence Interval (CI)
p = prevalence taken from the previous study as, 80%^7^
q = 1-p
e = margin of error, 9%

The minimum required sample size was 76. However, the final sample size taken was 79.

Overnight fasting blood samples were collected from the patient after 8-10 hours of fasting. Lipid profiles were measured where total cholesterol (TC), high-density lipoprotein cholesterol (HDL-C), triglyceride (TG) and low-density lipoprotein cholesterol (LDL-C) were calculated and measured.

Dyslipidemia is defined according to the third report of the National Cholesterol Education Program (NCEP) Adult Treatment Panel III. One or more of the following is diagnosed as dyslipidemia.^7^

1. LDL Cholesterol ≥130 mg/dl
2. Total Cholesterol ≥200 mg/dl
3. Triglycerides ≥150 mg/dl
4. HDL Cholesterol ≤40 mg/dl

Data were entered in Microsoft Excel 2016 and analysed using IBM SPSS Statistics version 21.0. The point estimate was calculated at a 95% CI.

## RESULTS

Among 79 patients with ischemic stroke, dyslipidemia was seen in 42 (53.16%) (42.09-64.23, 95% CI). High cholesterol was seen in 21 (50%) with mean total Cholesterol of 188.48±49.26 mg/dl, high triglyceride in 22 (52.38%) with mean triglyceride of 160.83±69.44 mg/dl, high LDL in 14 (33.33%) with mean LDL of 110.86±39.68 mg/dl, and low HDL in 20 (47.61%) with mean HDL of 43.33±8.68 mg/dl.

The mean age of the ischemic stroke patients presenting with dyslipidemia in this tertiary care centre was 60±14.93 years with a maximum of 87 years and a minimum of 20 years out of which 23 (54.76%) belonging to the age group less than 60 years and 19 (45.24%) were above 60 years.

Gender wise there was male predominance with males being 23 (54.76%) and females 19 (45.24%). A total of 15 (35.71%) males and 6 (14.29%) females had high total cholesterol, 12 (28.57%) males and 10 (23.81%) females had high triglyceride, 9 (21.43%) males and 5 (11.90%) females had high low-density lipoprotein, and 6 (14.29%) male and 14 (33.33%) female had low high-density lipoprotein (Table 1).

**Table 1:** Lipid profile among patients with dyslipidemia (n= 42).

Lipid Profile	Age		Sex		Total n (%)
	<60 years n (%)	>60 years n (%)	Male n (%)	Female n (%)	
High total cholesterol	12 (28.57)	9 (21.43)	15 (35.71)	6 (14.29)	21 (50)
High triglycerides	13 (30.95)	9 (21.43)	12 (28.57)	10 (23.81)	22 (52.38)
High LDL cholesterol	6 (14.29)	8 (19.05)	9 (21.43)	5 (11.90)	14 (33.33)
Low HDL cholesterol	10 (23.81)	10 (23.81)	6 (14.29)	14 (33.33)	20 (47.61)

Among 42 patients with dyslipidemia, 23 (54.76%) of the ischemic stroke patients were smokers, 12 (28.57%) of them were male and 11 (26.19%) were female. Whereas 10 (23.81%) were alcoholic, 10 (23.81%) were male and none of the ischemic stroke female with dyslipidemia were alcoholic. In our study 37 (88.10%) patient with 21 (50%) male and 16 (38.10%) female were having first episode of ischemic stroke (Table 2).

**Table 2:** Different characteristics of the ischemic stroke patients (n= 42).

Characteristics	n (%)	Sex	
		Male n (%)	Female n (%)
Smoking	23 (54.76)	12 (28.57)	11 (26.19)
Alcoholic	10 (23.81)	10 (23.81)	-
**Stroke type**			
First Episode	37 (88.10)	21 (50)	16 (38.10)
Recurrent	5 (11.90)	2 (4.76)	3 (7.14)

In our study 30 (71.43%) of the patient presented with weakness of the body in the form of hemiparesis, upper or lower limb weakness unilateral or bilateral, 29 (69.05%) presented with slurring of speech, 9 (21.43%) with facial deviation, 5 (11.90%) with headache, 3 (7.14%) with loss of consciousness and 3 (7.14%) with a tingling sensation. In this study, multi-morbidity was found in 23 (54.76%) patients having more than one disease. Hypertension (HTN) was the commonest risk factor with a total of 18 (42.86%) patients having been diagnosed with HTN, 13 (30.95%) had type 2 diabetes mellitus (T2DM) followed by 3 (7.14%) with cardiovascular disease (CVD), 3 (7.14%) had chronic obstructive pulmonary disease (COPD) and 1 (2.38%) with chronic kidney disease (CKD) (Table 3).

**Table 3:** Presenting complaints and comorbidity in ischemic stroke patient (n = 42).

Variables	n (%)	Sex	
		Male n (%)	Female n (%)
**Presenting complaints**			
Headache	5 (11.90)	3 (7.14)	2 (4.76)
Slurring of speech	29 (69.05)	15 (35.71)	14 (33.33)
Weakness of body	30 (71.43)	15 (35.71)	15 (35.71)
Facial deviation	9 (21.43)	7 (16.67)	2 (4.76)
Loss of consciousness	3 (7.14)	1 (2.38)	2 (4.76)
Tingling sensation	3 (7.14)	2 (4.76)	1 (2.38)
**Comorbidity**			
T2DM	13 (30.95)	8 (19.05)	5 (11.90)
HTN	18 (42.86)	10 (23.81)	8 (19.05)
CVD	3 (7.14)	-	3 (7.14)
COPD	3 (7.14)	-	3 (7.14)
CKD	1 (2.38)	1 (2.38)	-

## DISCUSSION

The prevalence of dyslipidemia among the ischemic stroke patients in our study was 42 (53.16%) with hypertriglyceridemia the most common finding constituting 22 (52.38%) which was similar to the finding from a study in our country 58.33%.^7^

Dyslipidemia is a well-known risk factor for cardiovascular and cerebrovascular events worldwide. Therefore, preventing and managing dyslipidemia can help to reduce the global burden of stroke.^8^ Unfortunately, there is limited information available on the prevalence, morbidity, and mortality of stroke in Nepal beyond urban areas, and no official reports have been published on the epidemiology of stroke throughout the country.^4^ However, changes in the epidemiological profile of stroke have been observed in clinical practice and confirmed by a study done in a similar setting.^6^ In developing countries such as Nepal, the incidence rate of stroke has increased by 100%, whereas in developed countries, it has decreased by 42% over the last four decades.^9^ This increase in stroke incidence is largely attributed to atherosclerosis, which is a major contributor to ischemic stroke.^10^ However, dyslipidemia is a modifiable risk factor for stroke, and therefore, its management can play a significant role in reducing stroke incidence.

Our findings of a 53.16% prevalence of dyslipidemia in ischemic stroke patients are consistent with a study conducted in northeast China, which found a prevalence of 62.1% in ischemic stroke patients.^10^ These results highlight the importance of identifying and managing dyslipidemia in stroke patients, particularly those with ischemic stroke, to reduce the risk of recurrent events and improve overall outcomes.

The relative risk of stroke associated with elevated serum cholesterol is high, as well as its population-attributable risk given the increase in incidence in elderly populations.^11^ A study in Japan among 2,351 Japanese inhabitants aged ≥40 years of age showed increased incidences of atherothrombotic and lacunar infarctions significantly with the increasing LDL cholesterol level, but no such associations were observed for cardioembolic infarction. Thus, it depicts a positive relation of increased LDL with small artery stroke as high LDL is usually implicated in atherosclerosis.^12^ In our study, the mean age of ischemic stroke associated with dyslipidemia was 60±14.93 years with most patients less than 60 years 23 (54.76%). A comparable age was also present in studies done in Nepal and India.^13^

One of the studies showed that men experience more ischemic stroke than females, but females experience more subarachnoid hemorrhage. In terms of stroke onset, women are on average 4 years older than men at the age of ischemic stroke.^14^ Our study shows the prevalence of ischemic stroke is more 23 (54.76%) in men than 19 (45.24%) in females, thus men have a higher prevalence of ischemic stroke than females, similar to the study done in a similar setting.^14^ The Asia Pacific Cohort Studies Collaboration found a 25% increase in ischemic stroke rates for every 1 mmol/L increase in total cholesterol.^15^ Similar study found the prevalence of dyslipidemia in the eastern part of our country, in a similar setting, to be 46.05%, with high triglyceride (TG) being the most commonly deranged parameter similar to the finding depicted by our study 22 (52.38%).^16^ Our study found that the prevalence of ischemic stroke was higher in patients with high TG levels and lower in patients with high levels of LDL.

Our study revealed the weakness of the body 30 (71.43%) in the form of hemiparesis of the upper limb or lower limbs to the commonest presenting complaint followed by slurring of speech 29 (69.04%) and facial deviation 9 (21.43%) similar to the finding done in a similar setting, which showed hemiparesis 59.16% as the commonest complaint followed by slurring of speech.^7^ We find 23 (54.76%) of the ischemic stroke patients to have multimorbidity with HTN being the commonest associate, which was quite less compared to the findings of a similar study.^17^

Ischemic stroke is multifactorial and requires various integrated approaches. So early screening, monitoring and intervention of the different risk factors as well as awareness will help in combating the burden of the disease as it is one of the leading causes of morbidity and disability in the Asian population. Therefore, a more substantial, in-depth, and multicentered prospective investigation should be conducted to examine the severity of the disease and its relationship with various variables in the context of the Nepalese community.

The findings from our study cannot reflect the general population because it was a single-centered study with a small sample size. This was a descriptive cross-sectional study so association with risk factors cannot be established.

## CONCLUSIONS

The prevalence of dyslipidemia in ischemic stroke patients was lower than the similar studies done in similar settings. Modifiable risk factors also play an important role in disease causation so timely action and knowledge regarding the risk factor can reduce the disease burden.
